# Impact of Coronary Function Testing on Symptoms and Quality of Life in Patients with Coronary Microvascular Dysfunction: Meta-Analysis of Randomised Controlled Trials

**DOI:** 10.3390/jcm14238461

**Published:** 2025-11-28

**Authors:** Temar Habtezghi, Adam Haq, Yanbo Jin, Nimrah Haq, Heerajnarain Bulluck

**Affiliations:** 1 Yorkshire Heart Centre, Leeds General Infirmary, Leeds Teaching Hospitals NHS Trust, Leeds LS1 3EX, UK; 2 Leeds Institute of Cardiovascular and Metabolic Medicine, University of Leeds, Leeds LS1 3EX, UK

**Keywords:** coronary microvascular dysfunction, angina with non-obstructive coronary arteries, ischaemia with non-obstructive coronary arteries, coronary function testing, microvascular angina, vasospastic angina, Seattle Angina Questionnaire

## Abstract

**Background/Objectives:** A significant proportion of patients with angina undergoing invasive coronary angiography have no obstructive coronary artery disease (ANOCA), often due to coronary microvascular dysfunction (CMD). Coronary function testing (CFT) enables the physiological endotyping of these patients during angiography. This meta-analysis aimed to evaluate whether CFT-guided therapy improves angina symptoms and quality of life compared with standard angiography-guided care. **Methods:** Major databases were systematically searched for randomised controlled trials (RCTs) up to September 2025. The primary endpoint was angina severity; secondary endpoints included angina limitation, stability, frequency, treatment satisfaction, and quality of life. Pooled analyses were performed using a random-effects model with inverse-variance weighting to derive the weighted mean difference (95% confidence interval, CI). **Results:** Three RCTs involving 535 patients (mean age 60 years, 64% female) met inclusion criteria. The disclosure of CFT results did not significantly improve overall angina severity (mean difference: 6.00, 95% CI −2.32 to 14.33; *p* = 0.16), with considerate heterogeneity (I^2^ = 92%). No difference was observed for angina frequency or quality of life. In contrast, angina limitation, stability, and treatment satisfaction all favoured the CFT-disclosed group, although the results were heterogeneous. **Conclusions:** Invasive CFT appears feasible and clinically relevant in patients with ANOCA. Although several SAQ domains improved following physiology-guided management, these findings require cautious interpretation given the modest sample size and considerable heterogeneity. Larger, methodologically robust trials are warranted to clarify whether a CFT-guided strategy should be routinely integrated into the diagnostic and therapeutic pathway for ANOCA.

## 1. Introduction

Coronary artery disease (CAD) includes a broad range of epicardial stenoses, ranging from flow-limiting to non-flow-limiting lesions. Contemporary data suggests that up to 70% of patients undergoing invasive coronary angiography (ICA) for angina have angina and non-obstructive coronary arteries (ANOCA) [1]. Within this broad group, a substantial proportion exhibits objective markers of myocardial ischaemia, termed ischaemia with non-obstructive coronary arteries (INOCA). In such cases, stable angina is often attributed to coronary microvascular dysfunction (CMD) [1].

The absence of obstructive lesions does not necessarily imply benign outcomes. Both ANOCA and INOCA are consistently linked to increased morbidity and mortality compared with individuals without CAD. Nevertheless, these conditions remain poorly recognised [2]. Challenges lie not only in the absence of standardised diagnostic algorithms but also in the tendency to dismiss symptoms as “non-cardiac chest pain” in clinical practice. Consequently, patients face recurrent hospitalisations and frequent emergency department visits, which adversely affect their quality of life [1].

Coronary function testing (CFT) now offers a means of interrogating microvascular and vasomotor function during ICA, and its diagnostic yield and safety profile have been well established [3]. CFT enables clinicians to identify distinct endotypes of ANOCA and INOCA, thereby supporting targeted therapeutic strategies rather than empiric treatment [4]. The 2023 AHA/ACC/ACCP/ASPC/NLA/PCNA guideline recognises invasive CFT as a promising but still investigational approach for suspected CMD, recommending its use only in selected centres [5]. In contrast, the 2024 ESC guideline grants a Class I recommendation for invasive CFT in patients with ANOCA/INOCA whose symptoms persist despite treatment, endorsing a mechanism-based diagnostic strategy that distinguishes microvascular from vasospastic endotypes to enable targeted therapy and improve outcomes [6]. This approach, which aims to improve angina and quality of life, has been the subject of recent randomised controlled trials (RCTs).

The CORMICA trial [7] reported that endotype-stratified therapy guided by CFT reduced angina severity and improved quality-of-life indices relative to angiography-directed management. By contrast, the CorCTCA trial [8] did not demonstrate a clear superiority of CFT-guided management. Therefore, we aimed to conduct a meta-analysis of RCTs to evaluate whether a CFT-guided management strategy improved angina symptoms and quality of life over an angiography-guided management strategy in patients with CMD.

## 2. Methods

This meta-analysis was performed following the Preferred Reporting Items for Systematic Reviews and Meta-analysis (PRISMA) framework [9].

### 2.1. Eligibility Criteria

We included all RCTs involving patients with ANOCA and compared operators that were blinded versus unblinded to the results of CFT, with a follow-up evaluation of angina symptoms. We excluded all studies that were not RCTs, published as abstracts/conference presentations only, or involved non-invasive coronary function testing.

### 2.2. Search Strategy

A systematic search was performed using the PubMed/MEDLINE, Cochrane Central, and Ovid/Embase databases to identify RCTs in English up to November 2025. The detailed search strategy is available in the Supplementary Materials. From the results, any duplicated, non-human participants and non-English-language studies were discarded prior to screening.

Two authors (AH, YJ) independently screened all 261 results obtained from the literature search by reviewing the titles and abstracts. Studies that were considered potentially relevant underwent a full-text review for eligibility and data extraction. Any discrepancies between the two authors in the study selection process were resolved by a third author (HB).

### 2.3. Data Extraction and Quality Assessment

Extracted data included study design, patient demographics, invasive coronary function testing (results disclosed vs. undisclosed to the operator), inclusion/exclusion criteria, impact of angina (assessed by the Seattle Angina Questionnaire), and follow-up period. The risk of bias for individual studies was assessed in detail using the Cochrane Risk of Bias 2 tool. We assessed the certainty of evidence for all outcomes using the GRADE approach, incorporating judgements on the risk of bias, inconsistency, indirectness, imprecision, and publication bias to generate an overall certainty rating for each domain.

### 2.4. Endpoints

The Seattle Angina Questionnaire (SAQ) was used in all 3 RCTs. There are 5 domains that are assessed in the SAQ, which includes angina limitation, angina stability, angina frequency, treatment satisfaction, and quality of life. The SAQSS averages the domains of angina limitation, frequency, and quality of life to provide an overall angina severity score. All SAQ domain scores and the SAQSS range from 0 to 100 with higher scores indicating less angina, fewer limitations, and better quality of life. The primary endpoint for this meta-analysis was the change in angina severity from baseline to 6 months’ follow-up. The secondary endpoints were the change in the 5 individual domains of the SAQ from baseline to 6 months’ follow-up: angina limitation, angina stability, angina frequency, treatment satisfaction, and quality of life.

### 2.5. Statistical Analysis

Statistical analyses were conducted using RevMan version 5.4 (Cochrane collaboration, Nordic Cochrane Centre, Copenhagen, Denmark). Clinical outcome data was extracted from each study. For trials reporting the median and quartiles, we used the median as a surrogate of the mean and the formula interquartile range/1.35 to derive the SDs [10]. Pooled analyses were performed using a random-effects model with inverse-variance weighting to derive the weighted mean difference. A positive mean difference, represented by a rightward shift on the forest plot, indicated a benefit in favour of CFT being disclosed to the operator, whereas a negative mean difference, represented by a leftward shift, indicated a benefit in favour of CFT not being disclosed. A two-sided *p*-value < 0.05 was considered to be statistically significant. Statistical heterogeneity was quantified using the I^2^ statistic, with thresholds of approximately 25%, 50%, and 75% representing low, moderate, and considerable heterogeneity, respectively, in accordance with the *Cochrane Handbook* [11]. Sensitivity analyses were conducted by sequentially excluding individual RCTs to assess the robustness and consistency of the overall effect estimate. If more than ten RCTs were available, publication bias was planned to be assessed visually using plots to explore potential asymmetry, which may indicate selective reporting or small-study effects.

## 3. Results

**Figure 1:**
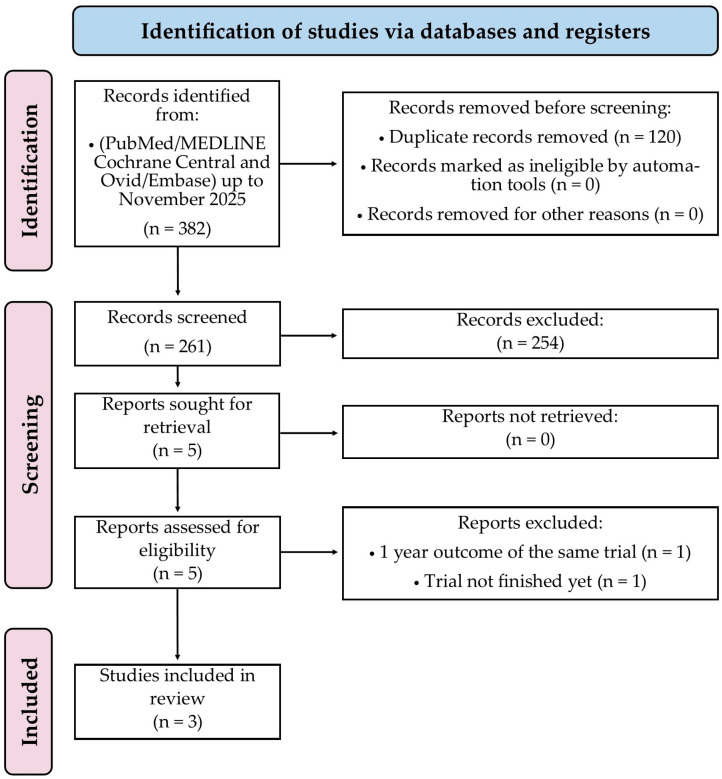
PRISMA 2020 flow diagram of study selection process. Flowchart summarising identification, screening, eligibility assessment, and inclusion of studies in this systematic review and meta-analysis. Three randomised trials met inclusion criteria.

A total of 382 records were found via database searches, of which 120 were excluded before screening, mainly due to duplication. The remaining 261 papers underwent title and abstract screening, and 3 RCTs [7,8,12] underwent full-text review and were subsequently included in this meta-analysis, as shown in Figure 1.

### 3.1. Characteristics of Included RCTs

A total of 535 patients were included across the three RCTs [7,8,12]. The CORMICA [7] and CorCTCA [8] trials were conceived and conducted at one centre in Scotland, whereas the ILIAS ANOCA trial [12] was a European multicentre trial (Table 1). The cohort had a mean age of 60 years, and 64% of participants were female. A total of 16% were diabetic, 62% had dyslipidaemia, 16% were current smokers, and 9% had experienced prior myocardial infarctions. Additionally, 47% of CorCTCA patients and 48% of ILIAS ANOCA patients had hypertension (not specified in CORMICA trial).

The CORMICA [7] and CorCTCA [8] trials reported their outcomes as the mean ± SD, and the follow-up was 6 months, whereas the ILIAS ANOCA trial [12] presented their outcomes as the median (25th quartile, 75th quartile), and follow-ups were at 6-month, 12-month, and long-term (≥18 months) periods.

### 3.2. Risk of Bias Assessment

**Figure 2:**
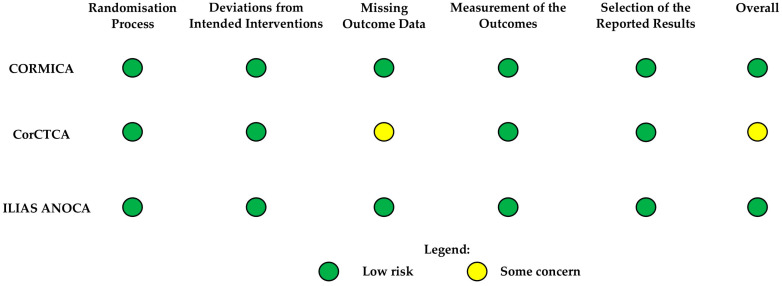
Risk of bias assessment for included randomised controlled trials. Traffic-light summary of risk of bias domains for each study (CORMICA, CorCTCA, and ILIAS ANOCA) based on Cochrane RoB 2 tool. Green = low risk; yellow = some concern; red = high risk.

The risk of bias assessment is shown in Figure 2. Two of the three trials—the CORMICA and ILIAS ANOCA trials [7,12]—were judged to be at a low overall risk of bias, reflecting rigorous trial design, effective blinding, robust outcome measurement using validated tools, and minimal missing data. In contrast, the CorCTCA trial [8] was rated as having some concerns, primarily due to potential biases introduced by missing outcome data during long-term follow-up, partially affected by the COVID-19 pandemic. Across all trials, the measurement of outcomes, primarily using the SAQ, was considered robust and unlikely to introduce bias.

A GRADE assessment (Supplementary Materials Table S1) of the three included RCTs indicated low certainty for the primary outcome of change in the SAQ summary score, primarily due to substantial statistical heterogeneity and imprecision of effect estimates. Certainty for secondary SAQ domains ranged from low to moderate, with treatment satisfaction receiving the highest certainty rating owing to the consistent direction of effect across trials.

### 3.3. Prevalence of CMD Endotypes

Across these three RCTs [7,8,12], the protocols for assessing both microvascular and vasospastic angina were not identical. ILIAS-ANOCA used a Doppler flow velocity wire (FloWire, Philips, The Netherlands) in the left anterior descending artery to derive coronary flow reserve (CFR) and hyperaemic microvascular resistance (hMR) during adenosine infusion at 140 µg·kg^−1^·min^−1^. CORMICA and CorCTCA relied on a pressure–temperature sensor wire (PressureWire X, Abbott, Westford, MA, USA) to obtain thermodilution-based CFR and the index of microvascular resistance (IMR). They defined dysfunction as CFR < 2.0 or IMR ≥ 25 and, importantly, assessed for microvascular spasm during acetylcholine (ACh) testing.

For vasospastic angina, all three groups followed the COVADIS diagnostic framework—≥90% epicardial constriction with angina reproduction and ischaemic ECG shifts—but the methodologies were not identical across the three RCTs [7,8,12]. ILIAS-ANOCA used manual intracoronary boluses of 2, 20, 100, and 200 µg into the left coronary artery, followed, if negative, by an optional 80 µg bolus to the right. CORMICA and CorCTCA favoured pump-driven infusions: both RCTs escalated concentrations of 10^−6^, 10^−5^, and 10^−4^ mol/L for two minutes each before a final bolus (100 µg left, 50 µg right).

Bearing these differences in mind, for the entire cohort, 228 (41%) patients were diagnosed with microvascular angina, 98 (18%) with vasospastic angina, and 73 (13%) with mixed microvascular and vasospastic angina. The remaining 28% had normal CFT results. Across all three contemporary CFT-guided RCTs [7,8,12], 72% of patients with angina and un-obstructive CAD had an identifiable CMD, with isolated microvascular dysfunction predominating.

Primary endpoint: Change in angina severity from baseline to 6 months.

**Figure 3:**
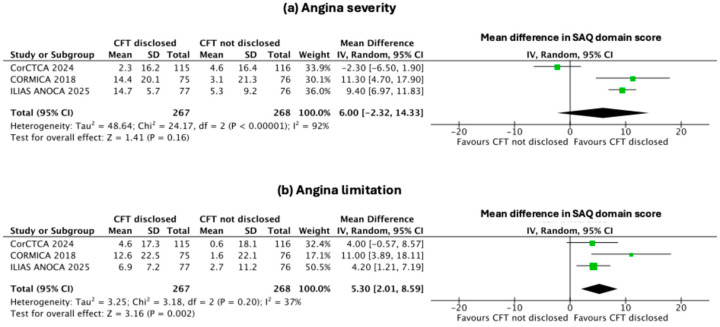
Effect of disclosure of invasive CFT results on angina severity and limitation. Forest plots comparing groups with CFT being disclosed versus not being disclosed for (**a**) angina severity and (**b**) angina limitation [7,8,12].

The disclosure of CFT to the operator did not result in a statistically significant improvement in angina severity compared with blinded management. The overall mean difference was 6.00 (95% CI –2.32 to 14.33; *p* = 0.16), with considerable heterogeneity (I^2^ = 92%), as shown in Figure 3a.

### 3.4. Secondary Endpoints: Change in Indices of Individual SAQ Domains

**Figure 4:**
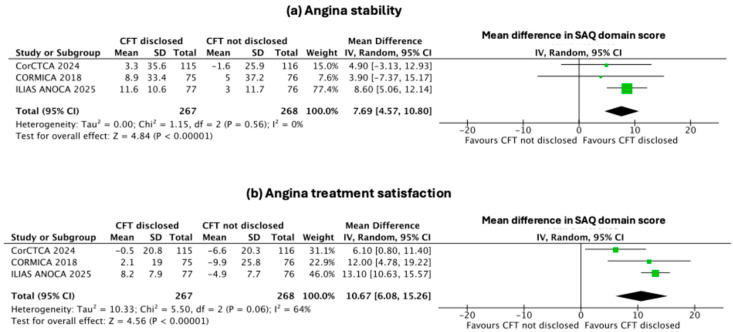
Effect of disclosure of invasive CFT results on angina stability and treatment satisfaction. Forest plots illustrating pooled mean differences for (**a**) angina stability and (**b**) treatment satisfaction. Results show significant improvement in both domains with CFT disclosure compared with non-disclosure [7,8,12].

**Figure 5:**
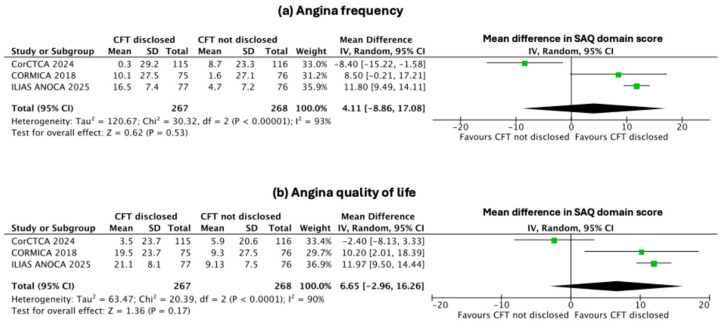
**Effect of disclosure of invasive CFT results on angina frequency and quality of life.** Forest plots showing pooled mean differences (random-effects model) for (**a**) angina frequency and (**b**) angina-related quality of life between groups with CFT results disclosed versus not disclosed [7,8,12].

The disclosure of CFT to the operator resulted in significant improvements in several secondary domains of the SAQ. The pooled mean differences (95% CI) favoured CFT disclosure for angina limitation [5.3 (2.01–8.59); *p* = 0.002, I^2^ = 37%, Figure 3b], angina stability [7.69 (4.57–10.80); *p* < 0.001, I^2^ = 0, Figure 4a], and treatment satisfaction [10.67 (6.08–15.26); *p* < 0.001, I^2^ = 64%, Figure 4b]. In contrast, improvements in angina frequency [4.11 (–8.86 to 17.08); *p* = 0.53, I^2^ = 93%, Figure 5a] and quality of life [6.65 (–2.96 to 16.26); *p* = 0.17, I^2^ = 90%, Figure 5b] were not statistically significant.

### 3.5. Sensitivity Analysis

To assess the robustness of the pooled estimates, a sensitivity analysis was performed by sequentially excluding one RCT at a time. Notably, the exclusion of the CorCTCA trial (thermodilution-based CFT), which contributed the largest sample size and the greatest heterogeneity, resulted in a statistically significant improvement in angina severity in favour of CFT disclosure (Figure S1). In contrast, the removal of either CorMicA (thermodilution-based CFT) or ILIAS-ANOCA (Doppler-based CFT) produced minimal changes in the pooled estimate. These findings suggest that the overall results are directionally stable and that the non-significance of the primary analysis was largely influenced by heterogeneity introduced by the CorCTCA trial.

## 4. Discussion

This meta-analysis integrated data from three contemporary RCTs—CORMICA [7], CorCTCA [8], and ILIAS-ANOCA [12]—to evaluate whether the disclosure of invasive CFT results to the operator improves angina severity and quality of life in patients with ANOCA. Collectively, these studies represent the current evidence base for physiology-guided management in this population.

Invasive CFT was associated with a favourable safety profile across all three trials. Serious procedural complications were rare, and no study reported excess major adverse cardiac events attributable to CFT. Transient symptoms during acetylcholine provocation were common but expected and self-limiting. Although safety reporting was not uniform enough for quantitative pooling, the overall dataset supports the notion that CFT is feasible and safe when performed in experienced centres.

Although the disclosure of CFT findings did not significantly improve overall angina severity, several domains of the SAQ, particularly angina limitation, stability, and treatment satisfaction, favoured physiology-guided management. These findings suggest that individualised therapy based on coronary physiology can translate into better symptom control and patient satisfaction, even when global composite scores show neutral results. The direction of effect was consistent across trials, and sensitivity analysis indicated that the neutral primary finding was largely driven by heterogeneity introduced by the CorCTCA trial [8], which enrolled a broader population and differed procedurally from the earlier CORMICA trial [7]. Several methodological features of the CorCTCA trial [8] help explain its contribution to heterogeneity [13]. Compared with the CORMICA trial [7], the CorCTCA trial [8] enrolled a broader population referred for CT angiography, used routine clinical follow-up rather than research-coordinated endotype-specific therapy adjustment, and applied a slightly different vasomotor testing protocol. These procedural and organisational differences likely diluted the treatment effect and contributed substantially to the observed heterogeneity in the pooled estimates.

In the subgroup analysis comparing thermodilution- and Doppler-based CFT, we did not observe any meaningful differences in treatment effect between the two physiological techniques. Although thermodilution was used in the CORMICA and CorCTCA trials, and Doppler exclusively in the ILIAS-ANOCA trial, the pooled estimates remained broadly comparable, and confidence intervals overlapped substantially. This suggests that variation in wire technology alone is unlikely to account for the heterogeneity observed in the primary analysis. Instead, differences in study design, follow-up intensity, and treatment implementation may have played a more dominant role. Given the limited number of trials, however, these findings should be interpreted cautiously.

### 4.1. Barriers to Clinical Application of CFT and Economic Implications

Despite its diagnostic value, the wider adoption of invasive CFT remains constrained by several practical barriers. Procedural standardisation varies across centres, acetylcholine availability is inconsistent, and the technique requires operators with specific expertise in both microvascular assessment and vasoreactivity testing. These factors, combined with longer procedural times and reimbursement variability, have limited uptake outside specialised programmes. Nevertheless, when performed within a structured pathway, physiology-guided care appears economically favourable. The dedicated health economic analysis from the CORMICA trial [14] reported an incremental cost-effectiveness ratio of GBP 4500 per QALY—far below the UK willingness-to-pay threshold of GBP 20,000 per QALY—with a >95% probability of being cost-effective. This estimate remained robust across sensitivity analyses and was driven primarily by clinically meaningful quality-of-life gains and reductions in repeat investigations. Taken together, although operational barriers persist, current evidence suggests that CFT-guided management is not only clinically informative but also economically sound, particularly when recurrent presentations and unnecessary angiography can be avoided.

### 4.2. Future Direction

The divergent results between the CORMICA [7] and CorCTCA [8] trials may, in part, stem from the nature of follow-up. CORMICA [7] participants were reviewed by a dedicated research team trained in CFT interpretation and therapy adjustment, ensuring adherence to endotype-specific management algorithms. The CorCTCA [8] trial, in contrast, relied on usual-care follow-up, where feedback to general cardiologists and primary care teams introduced greater variability. This distinction underscores that diagnostic precision alone is insufficient; without structured follow-up and reinforcement, therapeutic alignment may be inconsistent, attenuating observed benefit [13]. The iCorMicA trial (ClinicalTrials.gov NCT04674449) is designed with this insight in mind. As a large, pragmatic, multicentre study enrolling 1500 patients across Europe, it will evaluate physiology-guided care within real-world clinical follow-up frameworks rather than research-led environments. This design will test whether the benefits observed under idealised conditions in the CORMICA trial can be reproduced in routine clinical practice. The results will have direct implications for the scalability and sustainability of CFT-guided management in healthcare systems globally. Furthermore, disclosing CFT findings may facilitate a more precise stratification of subsequent therapies, an approach that aligns with emerging mechanistic data from the EDIT-CMD [15] and ChaMP-CMD [16] trials, where physiology-based endotyping guided targeted treatment pathways.

### 4.3. Emerging Non-Invasive Paradigms

The diagnostic and therapeutic management of suspected ANOCA increasingly requires an integrated approach that draws on both invasive and non-invasive modalities. Stress cardiovascular magnetic resonance (CMR), positron emission tomography (PET)-based flow quantification, and CT-derived flow indices can help pre-select patients before invasive assessment and refine the detection of microvascular dysfunction, while therapeutic strategies should be mechanism-based, ranging from endothelial-targeted therapies to anti-spasmodic regimens and structured risk factor optimisation, as emphasised by Taqueti and Di Carli [17]. Parallel to these invasive trials, the CorCMR trial [18] explores the use of cardiovascular magnetic resonance (CMR) as a non-invasive tool for coronary endotyping. By integrating perfusion mapping and T1/T2 mapping, CorCMR aims to identify microvascular and vasospastic phenotypes without the need for intracoronary instrumentation. Its results are anticipated to complement invasive CFT by defining which patients might be effectively “pre-screened” non-invasively before catheterisation. Together with the ongoing iCorMicA and CorCMR trials, these studies may point toward a tiered diagnostic pathway, combining precision, accessibility, and cost efficiency.

### 4.4. Strengths and Limitations

To our knowledge, to date, this is the first meta-analysis of randomised data investigating the benefit of coronary function testing. A major strength of this analysis is its exclusive focus on RCTs, eliminating the bias inherent in observational studies. Comparing groups in which CFT results were disclosed or blinded demonstrates with confidence that the benefits seen arose from acting on the results to guide therapy, rather than simply performing the test. The use of validated patient-reported outcome measures across all trials ensures that improvements were clinically meaningful and comparable. Furthermore, all three trials were conducted recently and are relevant to real-world clinical practice.

There are, however, several limitations of this study that should be acknowledged. Differences between the three trials extend beyond their follow-up structure. The ILIAS-ANOCA trial [12] employed a Doppler-derived CFR and hMR, typically yielding higher flow reserve values and fewer abnormal results, while the CORMICA and CorCTCA trials [7,8] used thermodilution-derived CFR and the index of microvascular resistance (IMR), a technique more sensitive to subtle microvascular dysfunction. Patient selection also differed: the ILIAS-ANOCA trial [12] required documented ischaemia before angiography, whereas the CORMICA and CorCTCA trials [7,8] recruited broader symptomatic ANOCA populations. For vasospasm testing, the ILIAS-ANOCA trial [12] used manual acetylcholine boluses, whereas the CORMICA and CorCTCA trials [7,8] employed infusion-based protocols. As Taqueti [19] emphasised, these procedural differences alter both the physiological provocation and the apparent prevalence of vasospastic angina. Our analysis was limited to trial-level rather than patient-level data. Interpreting these results against established thresholds of clinical relevance provides important context. A change of ≥5 points in any SAQ domain is generally considered the minimal clinically important difference [20]. In our analysis, the improvements observed in angina limitation and treatment satisfaction exceeded or approached this threshold, suggesting that these domain-specific effects may be meaningful from a patient perspective despite the neutral overall SAQ summary score. Even so, such signals should be regarded as exploratory given the modest sample size and heterogeneity across the included trials.

## 5. Conclusions

Overall, these findings suggest that invasive CFT could yield clinically valuable insights, improves patient experience, and is likely cost-effective, but this meta-analysis is limited by a small sample size and the heterogeneity of available RCTs. The ongoing iCorMicA trial will be pivotal in determining whether these benefits are maintained when care is delivered within standard clinical frameworks rather than research infrastructure. If successful, it will establish CFT-guided endotyping as both clinically effective and operationally feasible.

## Figures and Tables

**Table 1:** Characteristics of included trials.

Trial	Total Number of Patients and Country	Study Design/ Intervention	Diagnostic Method	Inclusion and Exclusion Criteria	Classification	Follow up Duration	Primary End-point
**CORMICA**	*n* = 151 United Kingdom	Randomised, open-label, stratified by invasive coronary function testing vs. usual care	Invasive coronary angiography with CFR, IMR (thermodilution method), and acetylcholine provocation testing	Inclusion criteria: • Adults with angina and non-obstructive coronary arteries (≤50% stenosis) • Positive stress test or clinical indication for invasive angiography Exclusion criteria: • Obstructive CAD (>50%) • Prior CABG or PCI • Significant LV dysfunction • Contraindication to adenosine or acetylcholine	Isolated microvascular angina (52%), isolated vasospastic angina (17%), mixed (21%)	6 months	Improvement in angina symptoms (SAQ score) comparing stratified vs. usual care
**CorCTCA**	*n* = 231 United Kingdom	Prospective, multicentre diagnostic study comparing CT-FFR and CT perfusion against invasive testing	CT angiography and invasive CFR, IMR (thermodilution method), and acetylcholine provocation testing	Inclusion criteria: • Adults referred for CT coronary angiography for stable chest pain • No flow-limiting stenosis Exclusion criteria: • Obstructive CAD (>50%) • Acute coronary syndrome • Prior revascularisation • Contraindication to adenosine or contrast	Isolated microvascular angina (55%), isolated vasospastic angina (12%), mixed (7%)	6 months (primary follow-up; additional analyses at 12 and ≥18 months)	Accuracy of CT-based functional testing (CT-FFR and CT perfusion) compared with invasive coronary testing
**ILIAS ANOCA**	*n* = 153 Netherlands and Germany	Multicentre, prospective registry evaluating invasive vasomotor testing in ANOCA patients	Invasive vasomotor testing with CFR, hMR (Doppler method), and stepwise acetylcholine provocation testing	Inclusion criteria: • Consecutive ANOCA patients with angiographically non-obstructive coronaries • Stable symptoms suitable for invasive testing Exclusion criteria: • Obstructive CAD (>50%) • Acute coronary syndrome • Major comorbidities limiting testing • Pregnancy • Contraindication to acetylcholine or adenosine	Isolated microvascular angina (7%), isolated vasospastic angina (42%), mixed (20%)	6 months	Prevalence and prognostic impact of invasive endotypes (CMD, vasospasm, mixed)

CFR: coronary flow reserve; IMR: index of microcirculatory resistance; ACh: acetylcholine; hMR = hyperaemic microvascular resistance; CMD: coronary microvascular dysfunction; FFR: fractional flow reserve; CT-FFR: computed tomography-derived fractional flow reserve; SAQ: Seattle Angina Questionnaire; ANOCA: angina with non-obstructive coronary arteries; CAD: coronary artery disease; CABG: coronary artery bypass graft; PCI: percutaneous coronary intervention; LV: left ventricle; ACS: acute coronary syndrome.

## Data Availability

The raw data supporting the conclusions of this article will be made available by the authors on request.

## Supplementary Materials

The following supporting information can be downloaded at: https://www.mdpi.com/article/10.3390/jcm14238461/s1: Search strategy; GRADE Evidence Profile for the Three RCTs (CORMICA, CorCTCA, ILIAS-ANOCA); Figure S1: Effect of disclosure of invasive CFT results on severity without CorCTCA trial; PRISMA Checklist.
